# Durable Response to Immunotherapy With Antiangiogenic Drug in Large-Cell Lung Carcinoma With Multiple Fulminant Postoperative Metastases: A Case Report

**DOI:** 10.3389/fonc.2021.633446

**Published:** 2021-05-20

**Authors:** Zhilin Luo, Hong Zhang, Yajie Xiao, Rui Wang, Liping Zhang, Chenglu Huang, Yu Cao, Chao Sun, Yongtian Zhao, Hanqing Lin, Dongfang Wu, Tianhu Wang

**Affiliations:** ^1^ Department of Thoracic Surgery, The Third Affiliated Hospital of Chongqing Medical University, Chongqing, China; ^2^ Department of Medicine, YuceBio Technology Co. Ltd., Shenzhen, China

**Keywords:** large-cell lung carcinoma, multiple postoperative metastases, loss of heterozygosity at HLA, immunotherapy, antiangiogenic drugs

## Abstract

Immunotherapy alone or chemo-immunotherapy has recently been recommended for treating advanced lung carcinoma in patients without driver mutations. However, the efficacy of immunotherapy and molecular mechanism in large-cell lung cancer (LCLC) remains unclear. Here, we reported a rare case of multiple fulminant postoperative body and mouth metastases in LCLC treating with combination immunotherapy. Initially, the patient was diagnosed as early stage LCLC and underwent a radical resection of the right lower lobe. Just one month later, multiple fulminant body and mouth lesions appeared in the right upper arm, right elbow, right waist, and tongue root. Meanwhile, serum neuron specific enolase (NSE) concentration dramatically increased from 12.12 to 30.14 ng/ml. Immumohistochemistry findings demonstrated moderate PD-L1 expressions with tumor proportion score (TPS), while next-generation sequencing indicated moderate tumor mutational burden (TMB) levels and gene mutations in *PBRM1* L1230P and *TP53* L194R of both foci. Besides, loss of heterozygosity (LOH) at human leukocyte antigen (HLA) class I (HLA-A*02:03, HLA-B*55:02 and HLA-C*12:03) were detected in the right upper arm metastasis, which may facilitate malignant postoperative metastases in this case. Notably, this patient received combination therapy with anti-PD-1 antibody sintilimab plus anlotinib, and achieved a partial response for at least 12 months. Using an integrated computational method, the mutant peptide TEIPENDIPL derived from PBRM1 L1230P was predicted to be a specific neoantigen and could still be presented by HLA-B*40:01. This case suggests that immunotherapy plus antiangiogenic drug may provide an alternative therapeutic option for advanced LCLC patients without common gene mutations.

## Background

According to the World Health Organization (WHO) guidelines, primary lung tumors can be classified into four major histological types: adenocarcinoma, squamous cell carcinoma, large-cell carcinoma and small-cell carcinoma (1, 2). Large cell lung carcinoma (LCLC) often happens in the periphery rather than in the center of the lung and can be distinguished with other lung cancers through pathological or immunohistochemical examinations (1, 2). At present, LCLC patients are individually treated based on divergent histological phenotypes and gene mutations (3). For example, actionable mutations in many common genes such as EGFR, ALK, KRAS, TP53 have been detected in some LCLC patients and provide the opportunities for appropriate targeted therapy accordingly (3).

Although platinum-based chemotherapy still served as the first-line standard therapy for those patients without targetable driver mutations (4), many patients have developed chemotherapy resistance and quickly achieved disease progression (5). To date, immunotherapy has brought in inspiring clinical outcomes in variety of cancer types, particularly for non-small cell lung cancer (NSCLC) (6). Also of note, immunotherapy alone or combination immunotherapy has recently been recommended for treating advanced lung carcinoma in patients without driver mutations (7). However, studies on the efficacy of immunotherapy in LCLC are very limited. Unlike other programmed cell death 1 (PD-1) inhibitors or programmed cell death-ligand 1 (PD-L1) inhibitors (nivolumab, pembrolizumab and atezolizumab) approved by America Food and Drug Administration (FDA), sintilimab has been approved in China for classical Hodgkin lymphoma (8, 9). Nevertheless, sintilimab alone or sintilimab plus chemotherapy has shown manageable toxicities and encouraging efficacies in several phase I–III clinical trials in NSCLC (stage IA–IIIB) patients in China (10–14).

In addition, there are still a subset of patients who cannot benefit from immunotherapies. Recent studies in cancer immunotherapy have shown that human leukocyte antigen (HLA) is of key importance for neoantigen presentations, which can activate the host immune system against pathogens and tumor cells (15, 16). Besides, loss of heterozygosity (LOH) at HLA highly involves with the immune escape mechanism in tumor evolution and may predict poor responses in anti-tumor immunotherapy in NSCLC (17, 18). Whereas, there are few reports on LOH at HLA in LCLC.

To the best of our knowledge, this is the first case reporting multiple fulminant postoperative body and mouth metastases in LCLC, which might be facilitated by LOH at HLA. Moreover, this patient received combinational therapy with anti-PD-1 antibody sintilimab plus anlotinib and achieved a partial response (PR) for at least 12 months.

## Case Presentation

**Figure 1:**
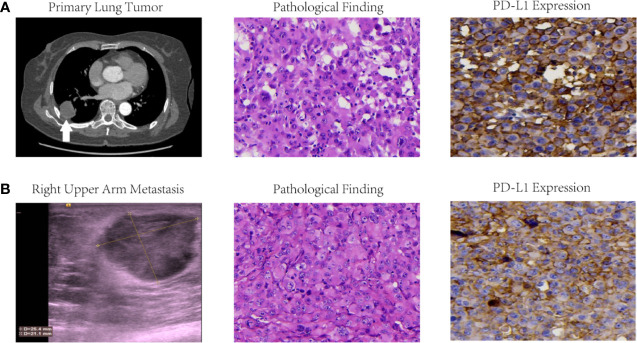
Imaging findings, pathological results and PD-L1 expressions of primary lung tumor **(A)** and right upper arm metastasis **(B)** in this LCLC case. Initial CT scan revealed a primary lung tumor in right lower lobe, and color ultrasound scan at 1 month after operation presented a right upper arm metastasis. Pathological results of both foci were confirmed LCLC with microscope magnification at 400×. Immumohistochemistry findings demonstrated PD-L1 expression with TPS 60–70% for the primary tumor and TPS 40–49% for right upper arm metastasis.

A 68-year-old Chinese female non-smoker presented with cough, bloody sputum and slight shortness of breath for more than 2 months and was hospitalized in June 2019. She had no other medical history or family history like hypertension, diabetes, or hyperlipidemia. Computer tomography (CT) (**Figure 1A**) revealed only a mass in the right pulmonary lower lobe and no bronchial or lymph node lesions. Then, she underwent a radical resection of the right lower lobe on June 2019. Pathological examination showed TTF-1(−), CgA(−), Syn(−), NapsinA(−), P40(−), CD5/6(−), CD56(−), MelanA(−), SOX-10(−), S100(−), HMB45(−), KI67 60%(+), CK(+), CKL(+), CKH(+). Taking together, she was initially diagnosed as stage Ib LCLC.

Unexpectedly, multiple fulminant body and mouth lesions was found in the right upper arm (**Figure 1B**), right elbow, right waist, and tongue root at 1 month after operation. Serum neuron specific enolase (NSE) concentration dramatically increased from 12.12 to 30.14 ng/ml. Further pathological examination of the surgical removal from the right upper arm presented CK(+), EMA(+), TTF-1(−), CgA(−), Syn(−), NapsinA(−), P40(−), CD5/6(−), CD56(−), Vim(+), KI67 80% (+), CD34(+), CD31(+), ERG(+). Therefore, she was confirmed stage IVb LCLC.

Immumohistochemistry staining demonstrated PD-L1 expression with tumor proportion score (TPS) of 60–70% for the primary tumor in right lower lobe (**Figure 1A**) and 40–49% for the body metastasis in the right upper arm (**Figure 1B**) respectively. The formalin-fixed paraffin-embedded (FFPE) tumor tissue slides together with matched blood samples were sent to College of American Pathologists (CAP)-authenticated biotechnique laboratory (Yucebio, Shenzhen, China) to perform next-generation sequencing (NGS) using YuceOne™ Plus extensive targeted panel for 1,021 genes. NGS data indicated tumor mutational burden (TMB) of 2.01 muts/Mb for the primary tumor in right lower lobe and 5.36 muts/Mb for the body metastasis in the right upper arm. Driver gene mutations in *PBRM1* L1230P and *TP53* L194R were found both in the primary tumor of the right lower lobe (variant allele frequencies of 2.65 and 5.14% respectively) and the body metastasis of the right upper arm (variant allele frequency of 63.18% and 57.96% respectively).

Besides, the primary tumor of the right lower lobe was genotyped as HLA-A*02:03, HLA-A*11:01, HLA-B*55:02, HLA-B* 40:01, HLA-C*12:03, and HLA-C* 07:02 using a modified computing method (19). According to another previous study (15), LOH at HLA-A*02:03, HLA-B*55:02 and HLA-C*12:03 were detected in the right upper arm metastasis while no abnormality was found at HLA-A*11:01, HLA-B* 40:01, and HLA-C*07:02. Moreover, the mutant peptide TEIPENDIPL derived from *PBRM1* L1230P was predicted to be a specific neoantigen using an integrated tool TrueNeo (20) and could still be presented by HLA-B*40:01 in both foci.

**Figure 2:**
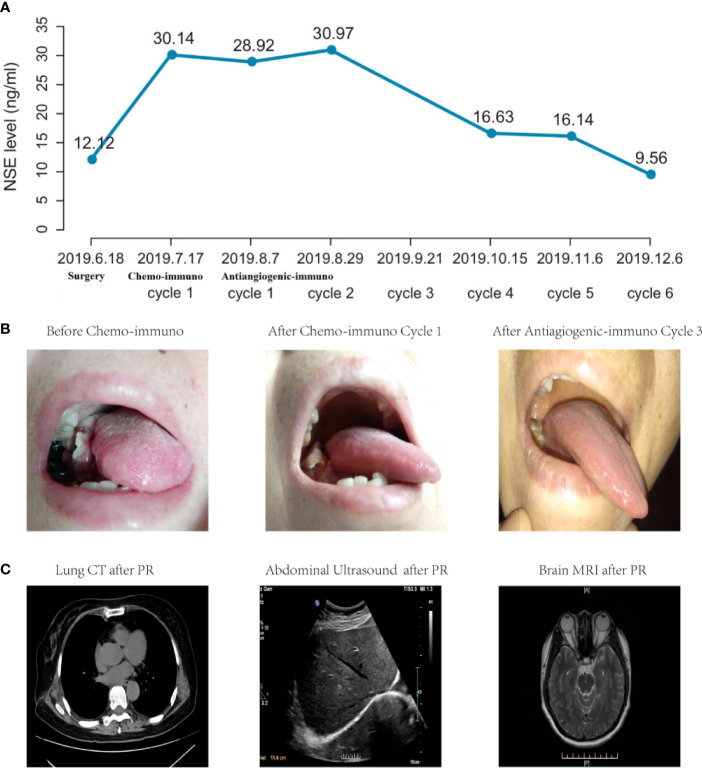
Clinical outcomes of NSE level **(A)**, mouth metastasis **(B)** and multiple organs **(C)** of this LCLC case. NSE level was detected before surgery or before each cycle treatment. After radical surgery, serum NSE level dramatically increased after surgery, and gradually decreased to normal levels after three cycle treatments of antiangiogenic-immunotherapy. In addition, mouth lesion appeared after surgery and disappeared after combination antiangiogenic-immunotherapy. Besides, there were no abnormity in multiple organs and PR lasted for more than 12 months.

Considering moderate expressions of predictive biomarkers for immunotherapy (PD-L1 expression levels <50% and TMB <10 muts/Mb) and fulminant metastases, this patient started chemo-immunotherapy with sintilimab (200 mg) plus etoposide (100 mg/m^2^, d1~3)-lobaplatin (50 mg/kg, d1) on July, 2019. Then, she suffered from several classical side effects of chemotherapy such as nausea, vomiting and fatigue, and refused to take any further chemotherapy. Although she developed a new lesion in left back and lymph node enlargement in left clavicle, there was no abnormity in the lung. Afterwards, she turned to another combination immunotherapy strategy of sintilimab (200 mg) plus multitargeted antiangiogenic agent anlotinib (12 mg) in August 2019. After 1-cycle treatment, multiple body lesions shrunk. Then, serum NSE level gradually decreased to 16.63 ng/ml (**Figure 2A**) and mouth lesion (**Figure 2B**) disappeared for 3-cycle continuous treatment in October 2019. In addition, there were no abnormity in multiple organs (**Figure 2C**). Besides, all the body lesions disappeared. The only remaining body lesion in the left back largely lessened and seemed like a fibrosis or scar in physical examinations. Thus, she achieved PR according to the therapeutic effects of all body and mouth lesions.

Based on the follow-up results, she has been tolerant without any unexpected side effects with combination immunotherapy and achieved a durable PR for at least 12 months till October 2020. Written informed consent was obtained from the patient for publication of this manuscript and any accompanying images. This patient is considering further lines of combination immunotherapy.

## Discussion

Immunotherapy with PD-1 inhibitors or anti-cytotoxic T lymphocyte antigen-4 antibodies, has emerged as a promising therapeutic strategy for patients with lung cancers. Unlike other common non-small cell lung cancers, potential molecular mechanism and therapeutic efficacy of immunotherapies in LCLC are still unclear.

Regarding moderate expression of predictive biomarkers for immunotherapy and no specific driver gene mutations, it was noteworthy that loss of HLA-A*02:03, HLA-B*55:02 and HLA-C*12:03 were detected in the right upper arm metastasis. Several recent studies have emphasized the important roles of cancer-specific neoantigens in determining cytolytic T cell activity and in predicting immunotherapy efficacy (21–23). Accordingly, downregulation of HLA genes or LOH at HLA may reduce antigen presentation and subsequently facilitate immune evasion (15, 18, 24). Lately, McGranahan et al. established a software tool to align sequencing reads with known human reference genome for a patient’s specific HLA type to calculate HLA LOH from 90 NSCLC patients from the TRACERx study and validated in 383 lung adenocarcinomas samples and 309 lung squamous cancer samples from TCGA database (15). They showed that HLA LOH happened more commonly in the branches instead of the trunk (15), suggesting that increased frequency of HLA LOH could enable tumor metastasis. In addition, they also demonstrated that HLA LOH may facilitate immune escape due to an active immune microenvironment (15). In consistent with their findings, we inferred that LOH at HLA-A*02:03, HLA-B*55:02 and HLA-C*12:03 may facilitate immune evasion, and subsequently enable multiple fulminant body and mouth metastases postoperatively in this case.

To date, PD-L1 expression has been recommended to help clinicians with choosing single-agent immunotherapy (TPS >50%) or chemo-immunotherapy (TPS 1–49%) (25). With the advances of NGS techniques, other biomarkers like TMB are emerged for predicting the efficacy of anti-cancer immunotherapy (26, 27). Besides, combination of PD-L1 expression and TMB has been presented to be related to reliable prognosis in advanced NSCLC patients following ICIs (28). Concerning moderate expressions of predictive biomarkers for immunotherapy (PD-L1 expression levels <50% and TMB <10 muts/Mb) and fulminant metastases, this patient started chemo-immunotherapy. After only one cycle of chemo-immunotherapy, she suffered from several classical side effects of chemotherapy such as nausea, vomiting and fatigue, and refused to take any further chemotherapy. Although she had a new lesion in left back and lymph node enlargement in left clavicle, there was no abnormality in the lung. Regarding no other common mutations like EGFR or ALK genes and patient willing, she turned to anti-PD-1 antibody sintilimab plus anlotinib and achieved a durable PR for at least 12 months. A recent phase 1b study presented good efficacy and safety of sintilimab and anlotinib in untreated NSCLC patients in China without EGFR/ALK/ROS1 mutations (29). Moreover, other clinical studies also demonstrated the synergistic antitumor effects of immune checkpoint inhibitors plus antiangiogenic drugs in a variety of solid tumors (30, 31). On the one side, antiangiogenic therapy exhibits the potential abilities to directly or indirectly alleviate immunosuppression and neoantigen presentation (32). On the contrary, immune checkpoint inhibitors prompt tumor vessel normalization (33).

Several clinical or genetic studies have recently highlighted correlations between tumor clonal neoantigens and the recognition of tumor cells by the immune system, which also has promoted growing preclinical and clinical interests in personalized vaccines and cell therapies targeted at these tumor clonal neoantigens (34, 35). Using a validated computational tool (20), we predicted the mutant peptide TEIPENDIPL derived from *PBRM1* L1230P to be a specific neoantigen presented by HLA-B*40:01 in both foci, which may provide a potential therapeutic target for personalized therapies. Beyond peptide-MHC binding affinity prediction, this prediction method considers multiple biological steps and provides prioritization of candidate high-immunogenicity neoantigens which have been tested *in vitro* and compared with other prediction method (20).

This study involved some expected limitations due to the lack of biological samples. At first, we cannot do any T cell infiltration experiment and HLA expression analysis to demonstrate the definite molecular association between HLA LOH in body metastasis and the immune escape mechanism in tumor microenvironment. Secondly, we are not able to test T cell responses with the specific neoantigen predicated using IFN-γ ELISPOT assay or other *in vitro* techniques. Further studies will be performed to better clarify potential molecular mechanism in LCLC and the therapeutic efficacy of immunotherapy plus antiangiogenic drugs in LCLC.

Although there are no definite guidelines for LCLC treatments, our clinical case suggests immunotherapy plus antiangiogenic drugs may provide additional therapeutic options for treating advanced LCLC patients without common gene mutations.

## Data Availability Statement

The original contributions presented in the study are included in the article/supplementary material Further inquiries can be directed to the corresponding author.

## Ethics Statement

Written informed consent was obtained from the individual(s) for the publication of any potentially identifiable images or data included in this article.

## Author Contributions

ZL, HZ, and YX acquired the data and prepared the manuscript. RW and LZ executed pathological and radiographic analyses. CH and YC interacted with the patient and did patient follow-up. CS, YZ, HL and DW performed data interpretation and revised the manuscript. TW supervised and revised the work. All authors contributed to the article and approved the submitted version.

## Conflict of Interest

YX, CS, YZ, HL and DW were employed by YuceBio Technology Co. Ltd.

The remaining authors declare that the research was conducted in the absence of any commercial or financial relationships that could be construed as a potential conflict of interest.
